# Cidofovir for the Treatment of Molluscum Contagiosum Virus

**DOI:** 10.3390/v14112484

**Published:** 2022-11-10

**Authors:** Erik De Clercq

**Affiliations:** Rega Institute for Medical Research, Herestraat 49, B-3000 Leuven, Belgium; erik.declercq@kuleuven.be

**Keywords:** acyclic nucleoside phosphonate (ANP), cidofovir, Molluscum Contagiosum (*M. contagiosum*), poxvirus

## Abstract

That cidofovir, an acyclic nucleoside phosphonate (ANP), was inhibitory to the replication of poxviruses was first demonstrated by De Clercq et al.. That its active metabolite, the diphosphate, was found to be inhibitory to the molluscum contagiosum (*M. contagiosum*) DNA polymerase was demonstrated by Watanabe and Tamaki. Twelve different independent observations have then indicated that cidofovir administered intravenously, topically or intralesionally is efficacious in the treatment of *M. contagiosum* mostly in immunosuppressed patients.

## 1. Introduction

In previous reviews, I have referred to the potential usefulness in the chemotherapy of poxvirus infections [1,2]. A new class of anti-poxvirus agents, that of the acyclic nucleoside phosphonates (ANPs), was heralded with the advent of (*S*)-HPMPA [(*S*)-9-(3-hydroxypropyl-2-methoxyphosphonyl)adenine] [3], soon to be followed by its cytosine counterpart (*S*)-HPMPC [(*S*)-1-(3-hydroxypropyl-2-methoxyphosphonyl)cytosine, cidofovir] [4]. The active metabolite of cidofovir, its disphosphate, was found to inhibit the vaccinia virus DNA polymerase [5]. Following a similar line of research, cidofovir diphosphate was then found to inhibit molluscum contagiosum DNA polymerase [6]. This opened the clinical use of cidofovir in the treatment of molluscum contagiosum.

## 2. Clinical Use of Cidofovir for Molluscum Contagiosum (*M. contagiosum*)

That cidofovir intravenous therapy or 3% cream led to the resolution of recalcitrant *M. contagiosum* virus lesions in HIV-infected patients was first demonstrated in 1997 by Meadows et al. [7]. Then followed the successful use of topical cidofovir 1% in the treatment of intractable *M. contagiosum* infection in a boy with Wiskott–Aldrich syndrome [8]. Zabawski and Cockerell [9] then reported the successful topical treatment of cidofovir 3% in Dermovan base in two children with *M. contagiosum*. Intravenous cidofovir therapy (5 mg/kg once a week followed by 5 mg/kg once every 2 weeks for maintenance therapy and probenecid (2 g, 3 h before the administration of cidofovir, and 1 g, 2 and 8 h later) effected the resolution of recalcitrant *M. contagiosum* lesions in an AIDS patient [10]. Topical 3% cidofovir has been advocated in the treatment of recalcitrant *M. contagiosum* in children infected with HIV-1 [11]. Intravenous cidofovir yielded a dramatic resolution of giant *M. contagiosum* in a patient with HIV [12]. Topical cidofovir 1% cream was found to be an effective and well-tolerated therapeutic option for the treatment of *M. contagiosum* in immunosuppressed children [13]. Intravenous cidofovir proved efficacious and safe in the treatment of giant *M. contagiosum* in an immunosuppressed patient [14]. Topical 1% cidofovir proved effective in the treatment of molluscum contagiosum in a patient with Jacobsen syndrome [15]. Recalcitrant *M. contagiosum* was successfully treated with intralesional cidofovir in a patient with HIV/AIDS [16].

Similarly, cidofovir 1% cream was considered to be an effective therapeutic alternative option for M. *contagiosum* lesions that are unresponsive to conventional methods in patients with HIV/AIDS [17]. Additionally, topical (1% or 3%) application of cidofovir was found to successfully treat a severe form of molluscum contagiosum in a patient treated for extensive atopic dermatitis with tacrolimus [18].

## 3. Conclusions

Edwards et al. [19] cautiously stated, as a 2020 European guideline on the management of genital molluscum contagiosum, that “immunosuppressed patients that develop severe and recalcitrant molluscum lesions may require treatment with cidofovir, imiquimod or interferon”. Leung et al. [20] postulated that for the treatment of *M. contagiosum*, physical destruction of the lesions, in particular, cryotherapy with liquid nitrogen and chemical destruction with cantharidin are the methods of choice for the majority of patients. They thereby cited a Cochrane systematic review in 2009, which showed that there was insufficient evidence to suggest superiority of any particular treatment [21]. A more updated systematic review was considered needed as there have been more studies showing the effectiveness of many therapeutic agents since the publication of the Cochrane systematic review (as cited by Leung et al. [20]). Here, I reviewed twelve independent observations [7,8,9,10,11,12,13,14,15,16,17,18], which altogether and albeit anecdotally, pertain to the usefulness of cidofovir in the (systemic, topical or intralesional) therapy of molluscum contagiosum.

There is a clear need for a well-conducted, controlled, adequately powered study to determine the efficacy of cidofovir (administered intravenously, intralesionally or topically) for the treatment of molluscum contagiosum in both immunocompromised and immunocompetent patients, possibly to be extended to other related poxviruses (i.e., mousepox) and other anti-poxvirus agents, i.e., brincidofovir (CMX001) and tecovirimat (ST-246).
